# Agricultural Commercialization and Dietary Transitions in Traditional Food Systems in Indonesia

**DOI:** 10.1016/j.cdnut.2026.109418

**Published:** 2026-06-30

**Authors:** Dominic Rowland, Ratna C Purwestri, Bronwen Powell, Mia Mustika Hutria Utami, Nia N Wirawan, Edi Waliyo, Maxi Lamanepa, Amy Ickowitz

**Affiliations:** 1 Center for International Forestry Research (CIFOR), Bogor, West Java, Indonesia; 2 Faculty of Forestry and Wood Sciences (FFWS), Czech University of Life Sciences, Prague, Czech Republic; 3 Institute of Nutritional Sciences, University of Hohenheim, Stuttgart, Germany; 4 Department of Nutrition, Faculty of Health Sciences, Universitas Brawijaya, East Java, Indonesia; 5 Department of Geography, Pennsylvania State University, University Park, PA, United States; 6 Management of Food Service and Nutrition Study Program, College of Vocational Studies, IPB University, Bogor, West Java, Indonesia; 7 Department of Nutrition, Poltekkes Pontianak, West Kalimantan, Indonesia; 8 Department of Nutrition, Poltekkes Jayapura, Jayapura, Indonesia

**Keywords:** nutrition transition, agricultural commercialization, livelihoods, cash crops, Indonesia, indigenous, diets, forests, palm oil

## Abstract

**Background:**

The impacts of agricultural commercialization on nutrition are highly contested. Agriculture–nutrition linkages are complex and context-dependent and may result in trade-offs between different dietary outcomes.

**Objectives:**

This study compared dietary patterns between children in oil palm and non-oil palm–adopting Indigenous households in two oil palm contexts in Indonesia: “plasma” smallholder oil palm in West Kalimantan and oil palm plantation labor in Papua.

**Methods:**

Using 24-h dietary recall data from children aged between 12 and 59 mo in West Kalimantan (696 children; 963 recalls across two seasons) and Papua (345 children), we compared quantities of foods consumed at the food group level between children in oil palm and non-oil palm Indigenous households. We also compared the probabilities of consuming ultraprocessed foods.

**Results:**

Children living in oil palm–adopting households in West Kalimantan consumed lower quantities of cereals and dark green leafy vegetables (DGLVs), more dairy and eggs, and were more likely to have consumed processed meat and sugar-sweetened beverages. In Papua, children living in households engaged in oil palm plantation labor consumed lower quantities of tubers and fish, but more cereals, DGLVs, and legumes. They were also more likely to have consumed ultraprocessed refined grains and bakery products.

**Conclusions:**

Associations between agricultural commercialization and children’s diets are context-dependent and may reinforce local dietary transitions. These may include increased consumption of some healthy foods in some contexts, but also be associated with diets linked to negative health outcomes, including reduced consumption of some nutrient-rich foods and increased consumption of ultraprocessed foods.

## Introduction

### Agricultural commercialization and diets

Agricultural commercialization has been widely promoted as part of efforts to “leverage agriculture for improved nutrition” [1,2]. However, despite its prominence in policy, the empirical evidence supporting this relationship is mixed and often inconclusive [[3], [4], [5], [6]]. The pathways linking agricultural change to nutrition outcomes are multiple, complex, and context-dependent [7,8]. The nutritional impacts of commercialization depend on baseline agricultural and market conditions and are mediated by gender-related differences in access to resources, control over income, and intra-household decision-making, resulting in heterogeneous outcomes across population [[9], [10], [11]]. Furthermore, agricultural changes do not typically occur in isolation, but unfold within broader livelihood transitions and concurrent social, economic, and food-system changes [[12], [13], [14], [15], [16], [17]].

In recent years, researchers have begun to focus on the contextual factors that mediate agriculture–nutrition linkages, as well as potential trade-offs between different dietary outcomes [6,18]. In some contexts, commercialization improves market access and increases food expenditure, thus improving diets [19,20]. Such associations appear strongest in contexts with well-functioning markets [[21], [22], [23], [24], [25]]. In other contexts, latent food preferences combined with market and food-system responses may increase consumption of unhealthy foods or reduce consumption of locally produced healthy foods [14,[26], [27], [28]]. Commercialization and specialization may also have adverse impacts on diets through reducing resilience to climatic or economic shocks [29,30], or through gender-mediated pathways such as gendered allocation of time [[31], [32], [33], [34]] or control over income and expenditure [29,35].

The lack of clear evidence on the effect of commercialization on diets and nutrition is further exacerbated by the lack of standardized dietary outcomes and the reliance on proxies of food security (e.g., calories) and nutrient adequacy (e.g., dietary diversity) [18,36]. Considerable debate surrounds the sensitivity of findings to different metrics [[37], [38], [39]]. Recent research indicates that agrarian change may affect the consumption of different food groups in different ways, resulting in nutritional trade-offs between different food groups, including potential adverse impacts on the consumption of unhealthy foods, overweight, and obesity [6,18]. As such, more granular data that capture the amounts of foods consumed, including ultraprocessed foods (UPFs), are needed to identify potential nutritional trade-offs.

### Oil palm, agricultural transitions, and diets in Indonesia

Oil palm development in Indonesia is highly controversial, and academic literature is routinely used and misused by both advocates and critics alike to further their claims [40,41]. Research over the past decade showing potential benefits of oil palm for economic development has allowed oil palm advocates to “construct a developmental narrative that positions the Indonesian palm oil sector as an indispensable force for good” [42]. Within this narrative, the purported benefits of oil palm for the food security and nutrition of local people have become a focal point [43,44]. Rowland [45] argues that this narrative has emerged through a combination of overgeneralizing findings from studies in a few specific oil palm contexts and a focus on limited dietary outcomes, as well the promotion of selective statistics by vested interests.

One of the difficulties in assessing the effects of oil palm production on diets and nutrition has been the homogeneity of the existing evidence. Existing evidence is based almost exclusively on expenditure survey data from one particular context—that of commercialized rubber farmers who adopt oil palm as independent smallholders in Jambi Province, Sumatra [45]. The generalizability of these findings, however, is debated [46,47]. Jambi Province has a unique market context based on its long history of plantation agriculture and transmigration, so that findings from this specific context may have “limited transferability” to other contexts [48]. To our knowledge, no previous dietary studies have been carried out in other oil palm contexts in Indonesia. Little is known about the potential impacts for those on the frontiers of oil palm expansion, who are more likely to be subsistence-oriented, rely more heavily on wild and forest foods, and who are engaging with oil palm via contemporary oil palm models and schemes [45]. In addition, little is known about the effects of oil palm engagement on overnutrition and the consumption of unhealthy foods—critical given the ongoing nutrition transition in Indonesia and the dramatic rise in the burden of diet-related noncommunicable diseases (NCDs) [49,50].

We investigate how oil-palm driven commercialization and market-oriented livelihoods are associated with dietary patterns across different contexts. We use the definition by Saha et al. [18] that agricultural commercialization is “the process of farmers becoming more integrated with markets of different kinds.” Market integration in this context does not exclusively refer to cash crops, but also to other forms of trade as well as wage labor [51]. Our aim was to capture associations between oil palm engagement and dietary intake among Indigenous communities in the regions where oil palm development is occurring most rapidly (West Kalimantan and Papua Provinces), focusing on the dominant contemporary model of oil palm encountered in these regions. These regions, like most in Indonesia [49], are already experiencing dietary transitions. Our focus, therefore, was on how dietary changes differ between communities engaging with oil palm and those following less commercialized livelihood trajectories.

## Methods

### Study design

Ideally, the study of transitions would use longitudinal cohort data to analyze changes within the same household or individual before and after the change. However, detailed dietary intake information was not available for Indigenous Indonesian communities experiencing agricultural change linked to oil palm to use as a baseline for the study. Therefore, we used a cross-sectional study design to compare the diets of children in households who worked in the oil palm sector with those of children in households who did not. We refer to these as oil palm (OP) and traditional livelihoods (TL), respectively. Households were considered to be OP if they had ≥1 member engaged in oil palm production and TL if they had no members engaged in oil palm production and either practiced noncommercial farming or primarily hunted and collected wild foods. Candidate households were those from Indigenous ethnicities with children aged between 12 mo and 12 y, but in this study, we focus on children aged <5 y. We received a list of households from the village health authorities that included ages of children and primary activities of their parents. We had planned to randomly select names from those lists to interview, but because of relatively small village populations, we surveyed all households that met the inclusion criteria in the 56 villages that we worked in.

There are likely endogenous factors influencing household or community adoption of oil palm, which could also be associated with dietary habits (see the section Study limitations for further discussion). Therefore, associations between OP and TL and dietary outcomes cannot definitively be causally attributed to oil palm. Likewise, non-oil palm–based livelihoods and diets continue to evolve—influenced by regional and local shifts in economies, opportunities, landscapes, and food systems, which may or may not be directly or indirectly influenced by oil palm development. As such, in neither site should comparisons between OP and TL diets be attributed solely to OP, nor can OP and TL groups be interpreted as reflecting “pre-” and “post-” oil palm. The study design, therefore, examines associations between different livelihood strategies and diets among households that historically shared similar landscapes and livelihoods but have diverged over time (see the section Study limitations). Although these associations do not permit definitive causal attribution, they help to characterize the nutritional landscapes of transitioning livelihoods. By identifying these patterns, the study provides empirical evidence of the dietary differences associated with distinct livelihood pathways, highlighting specific populations and nutritional indicators that may warrant targeted policy attention.

#### Study regions and site, village, and household selection

Our aim was to capture the associations between contemporary oil palm engagement and child dietary intake among Indigenous communities in the regions of Indonesia where oil palm development is occurring most rapidly. We therefore selected two provinces in Indonesia associated with the most rapid expansion of oil palm [52]—West Kalimantan and Papua.^1^ In both provinces, we focused on regions with the most recent history of oil palm expansion, and thus the district where oil palm most resembles contemporary models. The different nature of oil palm adoption in the two provinces necessitated different strategies for identifying OP and TL households and for selecting study villages, as described below.

#### West Kalimantan

In West Kalimantan (hereafter referred to as WK), we selected the district of Kapuas Hulu, which, before the arrival of oil palm expansion, was considered remote and inaccessible relative to other parts of Kalimantan [53,54]. Despite rapid palm oil expansion, the district remains relatively forested and includes two major national parks. As such, it is a key area of tension between environmental and economic development objectives [55,56].

The dominant contemporary model of oil palm in WK is that of smallholder “plasma” schemes – in which smallholders theoretically allocate part of their land to oil palm cultivation within a partnership arrangement with plantation companies, which provide capital, inputs, technical support, and processing and marketing services. Contemporary schemes in WK increasingly opt for “partnership” or “shareholder”-based variants in which plasma plots are integrated into the company estate [57,58]. Because plasma oil palm is adopted at the community level, the majority of residents within a plasma village are theoretically enrolled in a scheme. Consequently, mixed OP–TL villages are uncommon in Kapuas Hulu.

A list of candidate villages was first generated from all villages within Kapuas Hulu district using expert consultation and publicly available data. All candidate villages were required, at the baseline period (pre-2000), to have: *1*) predominantly Indigenous Dayak populations; *2*) swidden agriculture and forest-based livelihoods; and *3*) relatively similar access to markets. Focus groups were then carried out in each potential village, focusing on historical (pre-2000) livelihoods, demographics, and economic conditions. Livelihoods at the baseline period consisted of swidden agriculture combined with forest-based activities—hunting, fishing, and collection of nontimber forest products—as well as small-scale rubber agroforestry. No villages with extensive participation in logging or mining activities were included. Land tenure in all villages was historically based on customary land ownership. In addition, oil palm villages were required to have extensive community-wide participation in oil palm plasma schemes. Participants from both TL and OP households are from the Indigenous Dayak ethnic groups and therefore share similar food cultures.

#### Papua

Papua province is often viewed as the next “oil palm frontier” in Indonesia with vast tracts of forests and much lower population densities than the rest of Indonesia [52]. Traditional livelihood strategies for most Indigenous Papuans are largely based on hunting, fishing, and harvesting of wild foods, including sago, the traditional staple food, which is a starch extracted from the *Metroxylon* sp that grows wild in the region [59]. Consistent with other studies in the region [[60], [61], [62]], our scoping research indicated that commercial oil palm estates are the dominant contemporary form of oil palm in Papua; scoping research was unable to identify any plasma scheme models comparable with those in WK.

In Papua, OP households are typically engaged in wage labor on company estates and live in company barracks on plantations close to their villages. Thus, villages in Papua have both OP and TL households. Because both OP and TL households are present in the same villages, we compared the diets of children from OP and TL households originating from the same villages, noting that many OP households live part-time in nearby barracks located within the company oil palm estate. We received permission from the oil palm companies to interview those households in their barracks. We therefore compare the diets of children from OP and TL households who live in mixed OP–TL villages.

To select villages in Papua, we met with government officials from the agricultural ministry in Jayapura, the provincial capital, and obtained a list of locations throughout the province where there was commercial oil palm production. Using that list, we consulted key informants to identify villages near oil palm plantations where most residents were Indigenous Papuans. We visited the villages in a scoping exercise to check if they met the inclusion criteria. Several villages were eliminated because it was found that most of those who worked in oil palm were migrants. One site was eliminated because the oil palm company refused to let us interview their workers. Villages were included in the study if they met the following inclusion criteria: *1*) village residents were predominantly Indigenous Papuan ethnicities; and *2*) historical livelihoods as well as contemporary TL were based primarily on hunting, fishing, and foraging. OP households were Indigenous Papuan households where ≥1 member of the household worked for oil palm companies. In order to reach our target sample size we had to work in two districts – Merauke and Jayapura. All respondents were Indigenous Papuans; the ethnic group in the Merauke sample was Marind, and in the Jayapura sample, the Orya were the dominant ethnic group.

### Data collection

The study protocol was approved on 3 November, 2016, by the Ethics Committee of the Health Polytechnic Makassar, Indonesia, no. 302/KEPK-PTKMKS/XI/2016. Recruitment of enumerators began on 31 January, 2017. We obtained permission from Badan Kesatuan Bangsa dan Politik (the National and Political Unity Body) at the provincial and district levels, as well as Dinas Kesehatan (the Public Health Office) at district and subdistrict levels, to carry out our research. Prior, written informed consent was obtained from participants. Participants were informed that their names and other personal information would not be revealed. They were offered a small token gift (a small towel, bag, or soap) to thank them for their participation.

Data collection was coordinated by the provincial branches of the polytechnic schools of nutrition in the two provinces where the study was conducted—Poltekkes Pontianak and Poltekkes Jayapura. Ten enumerators were recruited for each site and received specialized training in dietary survey methods, including multiple-pass 24-h recalls, anthropometric measurements, and farming survey methods. This was followed by in-field testing and evaluation. Two supervisors accompanied the enumerators in the field at each site and were responsible for verifying survey completeness and providing continuous quality control throughout the data collection period (see Appendix E).

We received a list of households from the village health authorities in both sites that included the ages of children and primary activities of their parents. We had planned to randomly select names from those lists to interview, but because of relatively small population sizes, we in fact surveyed all households that met the inclusion criteria. The study was designed as an observational study of multiple dietary outcomes as part of a broader investigation of livelihood and landscape transitions. Our sampling strategy aimed to maximize statistical precision within the budgetary and logistical constraints. Given the the multi-outcome nature of the study and the absence of a single prespecified primary outcome, an a priori power analysis was not conducted. Instead, analyses focus on estimation, and results are presented as assotiations with 95% confidence intervals (CIs) to characterize the magnitude and precision of observed associations.

In WK, households were surveyed in 32 villages (TL=17, OP=15) across two seasons: a postharvest season (April–May 2017) and a preharvest season (October–November 2017). However, because of unusual rainfall patterns, some households had begun harvesting earlier than predicted and were thus excluded from the preharvest sample. Because of the high logistical and financial costs of fieldwork in Papua, data collection there was limited to a single season. We do not expect this to substantially affect the findings, however, because livelihoods in Papua are less seasonal than those in WK; traditional staple foods are sago and other tubers, which tend to be less seasonal than cereals, and forest-based activities are carried out year-round.

The dietary intake survey consisted of a 24-h multiple-pass dietary recall with female adult caregivers [63], which, in addition to the standard information on foods consumed, also included questions on the source from which each food was acquired by the household (own agricultural production, wild, purchased, or gifted). The survey also included basic demographic questions, as well as questions on farming practices, time use of female caregivers, and perceptions of health of females and children. Interviews and surveys typically took around one hour to administer. Respondents were asked about their own dietary intake and that of one of their children aged between 1 and 5 y. (In Papua, we included children aged ≤12 y in the survey, but in the current study we use data only from children aged ≤5 y). When there was more than one child in the target age range, we asked about the oldest child. Respondents reported portion sizes using a standard set of local units (e.g., spoons, cups, and bowls). These were converted to weights using conversion factors based on weighing local foods with these same units (see Appendix E). For mixed dishes, recipes including quantities of ingredients were recorded along with the total volume produced. During data analysis, ingredient weights were adjusted using yield factors to account for changes in food weight during preparation and cooking. The proportion of each recipe consumed by a respondent was calculated based on the reported amount consumed relative to the total recipe yield. This proportion was then applied to the yield-adjusted ingredient weights to estimate the amount of each ingredient consumed.

The final WK sample consists of 696 children across 32 villages in Kapuas Hulu district (TL = 342, OP = 354), of which 321 children were surveyed across both seasons (TL = 166, OP = 155) and a total of 375 surveyed in only 1 season (TL = 176, OP = 199). The final Papua sample consists of 378 children across 24 villages and 2 districts (TL = 201, OP = 177).

### Analysis

Table 1 shows the summary statistics for participating households in the study. Eleven households in WK declined to be surveyed for a second time in the postharvest season. Incomplete 24-h recalls (WK: *n* = 1 preharvest, *n* = 2 postharvest) were excluded before analysis. In addition, missing covariate data resulted in Stata dropping observations from the model. In WK, 54 observations were excluded because of missing wealth-quintile (asset-index) data. In Papua, 33 children were excluded due to missing wealth-quintile (asset-index) data. We compared children’s food consumption in OP and TL households in the WK and Papua sites, controlling for differences in individual and household characteristics. All analyses were performed in Stata 17 (StataCorp). For unprocessed food groups—cereals, dairy, eggs, fish, legumes, green leafy vegetables (GLVs), other vegetables, meat, fruits, and tubers—we used ordinary least squares (OLSs) regressions to model the associations between amounts of each food group consumed within 24 hours preceding the survey on household and participat_i_on in oil palm and a range of control variables given by the equation:

(1) $${Foodgroup}_{i,k}=\beta_{0}+\beta_{1}{OP}_{i}+X_{child}\beta_{\mathrm{child}}+X_{caregiver}\beta_{\mathrm{caregiver}}+X_{hh}\beta_{hh}+\beta_{5}{SEASON}_{i}+\epsilon_{i}$$

where the dependent variable is the grams of food from food group k consumed by child i in the 24 hours preceding the survey, and OP indicates whether the child is from an OP or TL household. The vector CHILD includes individual characteristics (age and sex of child), whereas the household and female caregiver’s characteristics are captured in the vectors CAREGIVER and household. Female caregivers’ characteristics included their age, educational status, and whether they originated from the surveyed village. SEs were estimated, accounting for the clustering of individuals within villages, using cluster-robust SEs in Stata. In Papua, we also included a variable for the female caregiver’s ethnicity. Household characteristics included the number of children in the household as well as the wealth quintile of the household constructed using an asset-index approach based on the article by Filmer and Pritchett [64]. The list of assets used to construct the index is shown in Appendix A. For WK, where >1 season of data were collected, we also included a binary variable for the season in which the sample was taken. As is common with dietary data, the food group consumption was right-skewed in both sites (WK: skewness range depending on food group 1.7–28.0; Papua: 1.3–16.8). OLS is robust to nonnormality of the outcome variable, but we assessed the sensitivity of our findings to these distributional features by repeating all food-group regressions using log-transformed outcomes (ln[x+1]) as a sensitivity analysis.

**TABLE 1:** Characteristics of the analytic sample, by livelihood group

Characteristic	West Kalimantan		Papua	
	TL (*n* = 480)	OP (*n* = 483)	TL (*n* = 186)	OP (*n* = 159)
Age group (y), *n* (%)				
1–2	96 (20.0)	90 (18.6)	37 (19.9)	30 (18.9)
2–3	93 (19.4)	128 (26.5)	41 (22.0)	43 (27.0)
3–4	135 (28.1)	119 (24.6)	47 (25.3)	30 (18.9)
4–5	156 (32.5)	146 (30.2)	61 (32.8)	56 (35.2)
Female, *n* (%)	269 (56.0)	234 (48.4)	92 (49.5)	85 (53.5)
Wealth quintile, *n* (%)				
Quintile 1	100 (20.8)	136 (28.2)	41 (22.0)	33 (20.8)
Quintile 2	114 (23.8)	97 (20.1)	102 (54.8)	31 (19.5)
Quintile 3	106 (22.1)	58 (12.0)	0 (0.0)	65 (40.9)
Quintile 4	65 (13.5)	97 (20.1)	16 (8.6)	0 (0.0)
Quintile 5	95 (19.8)	95 (19.7)	27 (14.5)	30 (18.9)
No. of children in household, median (IQR)	1.0 (1.0–2.0)	1.0 (1.0–1.0)	2.0 (1.0–3.0)	2.0 (1.0–3.0)
Caregiver education, *n* (%)				
Primary	405 (84.4)	448 (92.8)	98 (52.7)	76 (47.8)
Middle	23 (4.8)	13 (2.7)	41 (22.0)	38 (23.9)
Secondary	52 (10.8)	22 (4.6)	47 (25.3)	45 (28.3)
Caregiver born outside village, *n* (%)	323 (67.3)	294 (60.9)	159 (85.5)	117 (73.6)
Survey season, *n* (%)				
Preharvest	238 (49.6)	241 (49.9)	—1	—1
Postharvest	242 (50.4)	242 (50.1)	—1	—1
Ethnicity, *n* (%)				
Marind	—1	—1	111 (59.7)	86 (54.1)
Yei	—1	—1	31 (16.7)	18 (11.3)
Other	—1	—1	44 (23.7)	55 (34.6)

Abbreviations: OP, oil palm household; TL, traditional livelihood household.

West Kalimantan figures are at the child-recall level (963 recalls from 696 children surveyed across 2 seasons); Papua figures are at the child level (345 children).

1 Not applicable/collected at that site.

For ultraprocessed foods (UPFs), we did not use the amount in grams because of the heavily zero-inflated data distribution, but instead modeled the probability of a child having consumed ≥1 UPF using a logit regression. Foods were categorized as UPFs if they met the definition of ultraprocessed (category 4) using the NOVA classification system [65]. We also subdivided UPFs into categories, including processed meats, sugar-sweetened beverages (SSBs), refined grain and bakery products, and sweets, and modeled each separately.

The econometric specification for the probability of consuming processed food is given by the equation:

(2) $$\mathrm{Pr}\;\left({UPF}_{i,j}=1\right)=\Lambda \left(\beta_{0}+\beta_{1}{OP}_{i}+X_{child}\beta_{\mathrm{child}}+X_{caregiver}\beta_{\mathrm{caregiver}}+X_{hh}\beta_{hh}+\beta_{5}{SEASON}_{i}\right)$$

where UPFi,j is a binary variable, and *j* goes from 1 to 5, indicating whether the child consumed any UPF or 1 of 4 subgroups of UPFs in the 24 hours preceding the survey, and the independent variables are the same as those used in Equation *1*.

## Results

### Dietary intake of nonprocessed food groups

**FIGURE 1:**
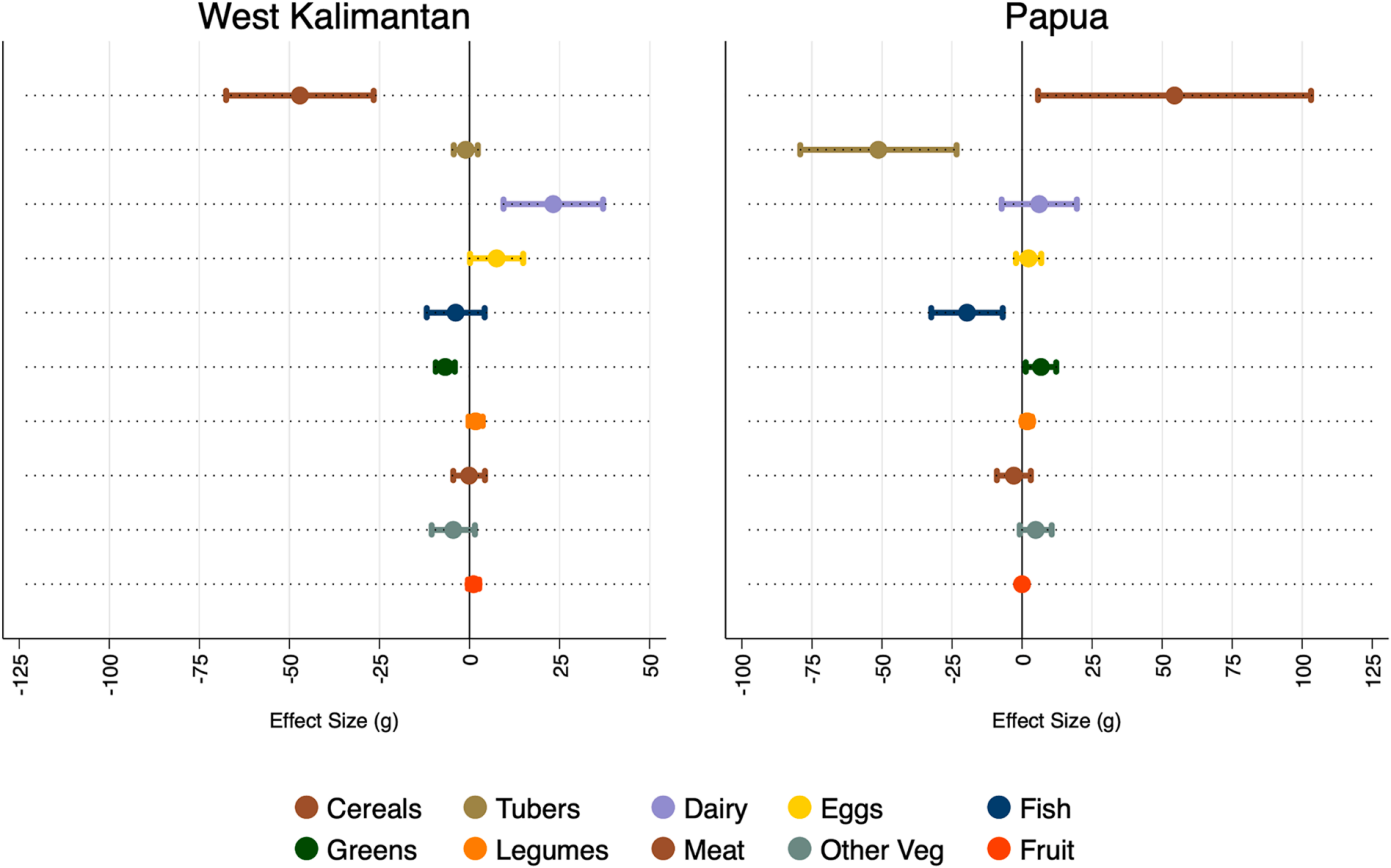
Estimated associations between household oil palm engagement and children's consumption of unprocessed food groups. Values are OLS coefficients (adjusted difference in grams/day) for children in oil palm compared with traditional livelihood households. Confidence intervals at the 95% level.

Figure 1 shows the estimated differences in associations children’s intake of different (nonprocessed) food groups associated with being in an OP household compared with a TL household, adjusting for household, child, female caregiver, and site characteristics. Coefficients, CIs, and cluster-robust SEs for all independent variables are reported in Appendix B.

In the WK sample, children in OP households consumed 44.4 fewer grams of cereals (*P* < 0.01; 95% CI: –66.3, –22.6), 7.1 fewer grams of dark GLVs (DGLVs) (*P* < 0.01; 95% CI: –9.9, –4.3), but 24.6 more grams of dairy (*P* < 0.01; 95% CI: 10.8, 38.3) and 8.3 more grams of eggs (*P* < 0.05; 95% CI: 1.3, 15.2) in the 24 hours preceding the survey than children in TL households. All other food-group differences were not statistically significant. In the Papua sample, children in the OP group consumed 54.4 more grams of cereals (*P* < 0.05; 95% CI: 5.7, 103.2), 6.8 more grams of DGLVs (*P* < 0.05; 95% CI: 1.3, 12.2), and 1.9 more grams of legumes (*P* < 0.05; 95% CI: 0.1, 3.6), but 19.7 fewer grams of fish (*P* < 0.01; 95% CI: –32.4, –6.9), and 51.3 fewer grams of tubers (*P* < 0.01; 95% CI: –79.2, –23.4) in the preceding 24 ho than children in TL households.

The direction and statistical significance of the main findings were largely unchanged in the log-transformed sensitivity analyses (Appendix D). In WK, statistically significant associations with cereal, dairy, egg, and DGLV consumption were robust to the transformation. In Papua, statistically significant associations between oil palm engagement and cereal, fish, legume, and tuber consumption remained significant after log transformation. The positive association with DGLV consumption in Papua did not survive the transformation and should therefore be interpreted with caution.

### Dietary intake of UPFs

**FIGURE 2:**
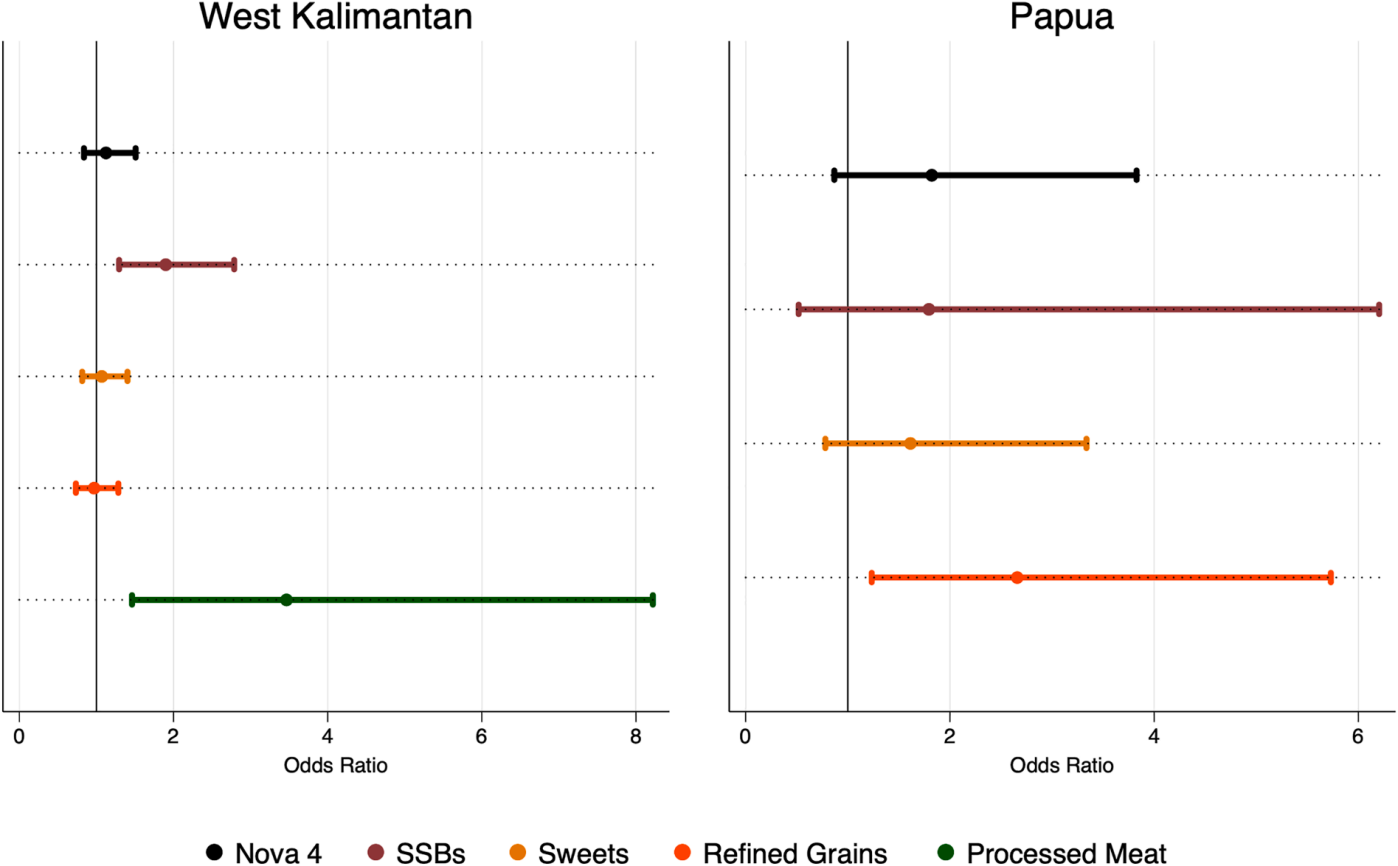
Adjusted odds ratios for asscEstimated associations between household oil palm engagement and children's consumption of processed food groups. Values are odds ratios from logistic regression for the probability of a child having consumed ≥1 item in each category (oil palm compared with traditional livelihood households). Confidence intervals at the 95% level. SSB, sugar-sweetened beverage.

Figure 2 shows the adjusted odds ratios for the association between household oil palm (OP) engagement, compared with traditional livelihoods (TL), and children's consumption of different ultra-processed food categories. Coefficients, CIs, and cluster-robust SEs for all independent variables are reported in Appendix C. In WK, children in OP households were >4 times as likely to have consumed processed meat products as children in TL households [odds ratio (OR): 4.02; 95% CI: 1.61, 10.03; *P* < 0.01] and were also significantly more likely to have consumed SSBs (OR: 1.74; 95% CI: 1.18, 2.57; *P* < 0.01). No significant differences were observed between OP and TL groups for UPFs overall, sweets, or refined grain and bakery products.

In Papua, children in OP households were more than 2 and a half times as likely (OR: 2.66; 95% CI: 1.23, 5.73) to have consumed ultraprocessed refined grain products (e.g., instant noodles and bakery products) in the preceding 24 hours as children in TL households (*P* < 0.05). Although higher consumption was observed for other UPFs (NOVA 4 and SSBs), these differences were not statistically significant.

### Food sources and dietary patterns

**FIGURE 3:**
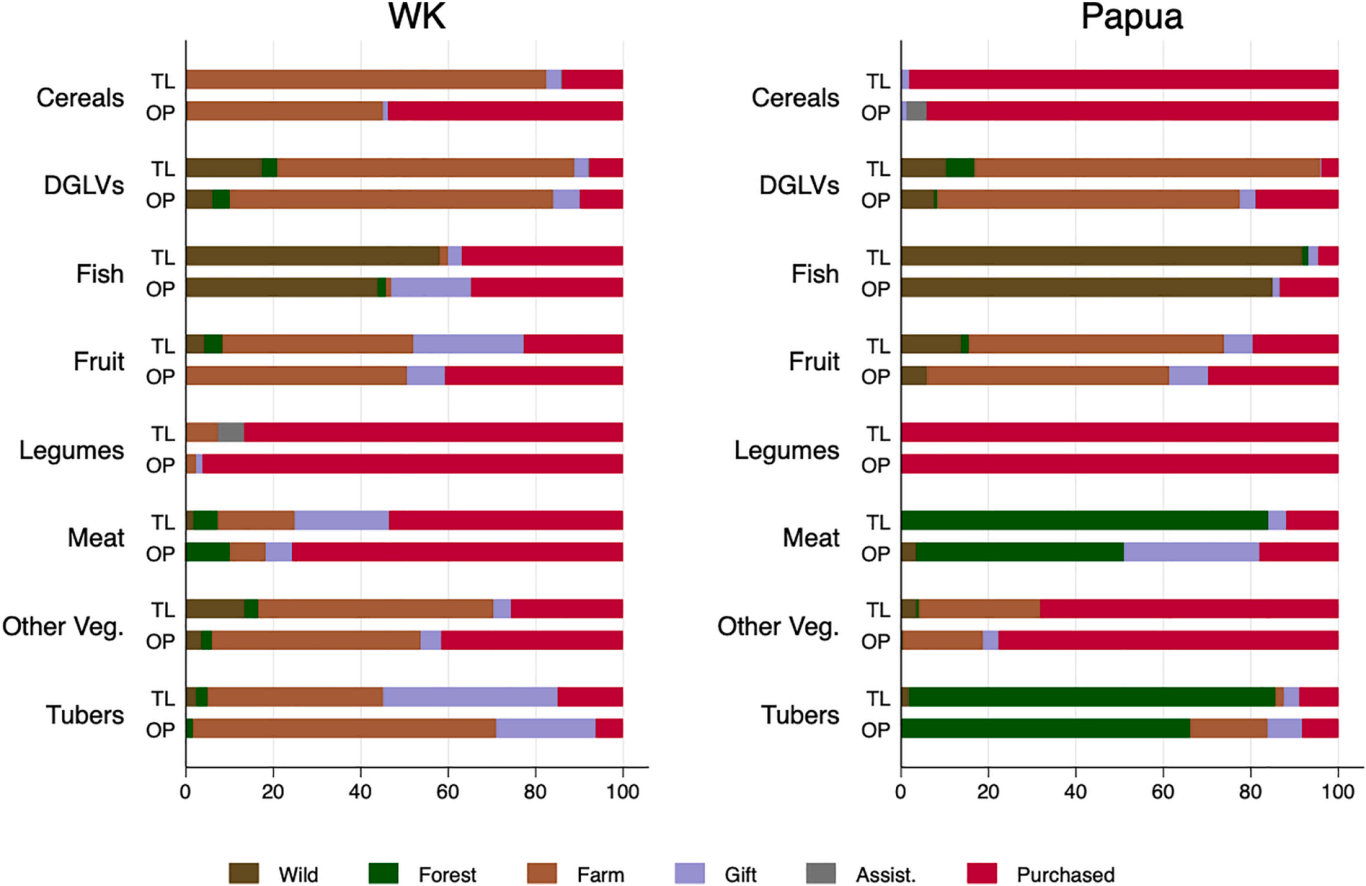
Proportion of foods consumed within each food group coming from different sources (by weight), by livelihood group and site.

Figure 3 shows the proportion of foods within food groups (by weight) acquired from different sources. Overall, children in OP households consumed significantly more purchased foods (by weight) in both sites, with children in OP households consuming almost double the amount of purchased foods compared with those in TL households in WK and one and a half times more in Papua (Papua: TL = 50.6%, OP = 75.8%, *P* < 0.001; WK: TL = 38.0%, OP = 67.9%, *P* < 0.001). In both sites, children in OP households obtained a smaller share of fish, fruit, DGLVs, and other vegetables from wild sources than those in TL households. For each of these food groups (with the exception of fish in WK), the share of consumption coming from purchased sources was also higher in the OP site.

The share of foods coming from different sources indicates different patterns of food acquisition between TL and OP groups. For instance, in WK, markets are the main source of meat in both TL and OP, although these are also obtained from wild sources and as gifts. In contrast, in Papua, wild sources (predominantly forests) are the most important source of meat in both OP and TL, making up 84% of meat consumed in the TL group and just over half (51%) in the OP group. Interestingly, the proportion of meat consumed that was gifted in Papua is around seven times higher in OP than in TL (TL = 4.1%, OP = 30.1%). Forests and other wild food environments were important sources of fish and tubers (predominantly sago) for both groups in Papua.

## Discussion

Previous studies of oil palm and diets have focused on “independent” smallholders in one province of Indonesia (Jambi) [66], a province oil palm (and plantation agriculture more generally) has a long history and in which local people grow little of their own food [45]. These studies looked at various measures of nutrition including dietary diversity, micronutrient adequacy, and calorie consumption estimated based on seven-day household-level recall data [66] but did not address consumption of potentially unhealthy foods. Overconsumption and the consumption of UPFs are rapidly becoming one of the major contributors to malnutrition and diet-related NCDs in Indonesia. We therefore analyze associations between oil palm and the dietary intake of both healthy and unhealthy food groups using validated dietary intake survey methods. Given that associations between oil palm and social, economic, and dietary outcomes are highly context-dependent [48], we do so in two different contexts on the frontiers of oil palm expansion in Indonesia.

### Dietary differences between oil palm and traditional livelihoods

In both the Papua and the WK samples, children in OP-engaging households have different diets compared with those of children in households with more TL—even after controlling for potential confounding factors. The observed differences are mixed with respect to dietary quality: OP children consumed lower quantities of some healthy food groups, higher quantities of others, and higher quantities of some unhealthy food groups.

Children in the OP group in WK consumed less food from healthy, nutrient-dense food groups including DGLVs (*β* = –7.10 g; *P* < 0.01; 95% CI: –9.89, –4.31) but consumed more dairy (*β* = +24.6 g; *P* < 0.01; 95% CI: 10.81, 38.30) and eggs (β = +8.3 g; *P* < 0.05; 95% CI: 1.31, 15.23), which are two nutrient-rich food groups especially important for young children’s growth and development [67,68]. In addition, although children in the OP group in WK consumed similar quantities of meat, ultraprocessed meat made up a greater share of consumption (OP = 29%, TL = 8%, *P* < 0.001). Children also consumed more SSBs. The increased share of processed meat may result in adverse health impacts. The International Agency for Research on Cancer classified processed meat as a group 1 carcinogen, which means that there is strong evidence that processed meat causes cancer [69,70]. Studies from Indonesia have shown associations between consumption of processed meat and all-cause mortality [50]. It is also important to interpret the lower consumption of cereals in the OP group at the WK site in this context. High amounts of white rice consumption in Indonesia have been associated with increased risk of a range of diet-related NCDs such as type 2 diabetes [50,71]. However, locally consumed rice is a mix of white market rice and locally produced indigenous varieties of white and brown rice, with the latter likely to be nutritionally superior to the former [72]. As our data do not distinguish between the varieties of rice, it is not possible to predict the net-nutritional effect of the differences in rice consumption.

In the Papua site, the nutritional associations with oil palm also appear mixed, with some positive and some negative impacts. Children in OP households consumed less fish (*β* = −19.7 g; *P* < 0.01; 95% CI: –32.41, –6.90), but more DGLVs (*β* = +6.8 g; *P* < 0.05; 95% CI: 1.32, 12.19) and small amounts of legumes (*β* = +1.9 g; *P* < 0.05; 95% CI: 0.13, 3.58). Although the positive DGLV association in Papua did not remain significant after log-transformation (Appendix D) and so should be interpreted with caution, the legume association did remain significant. The latter is mostly explained by tofu and tempeh consumption (although total legume consumption was modest). In addition, children in OP households consumed more ultraprocessed refined grain products. However, the main difference between the diets in the OP and TL groups was the lower consumption of tubers (which is 98% sago in TL) and higher consumption of cereals (white rice) as the main staple food. Unlike in WK, where rice is a traditional food and many varieties are locally produced, in Papua, rice has only recently become a staple food and tends to be a hybrid commercial variety of rice that is purchased at a subsidized price [49]. The nutritional effects of this transition may be mixed, with rice being higher in protein and vitamins B1 and B3 but also lower in fiber and with a higher glycemic index [73]. As such, the rise in rice consumption in Papua over the past few decades is thought to be a contributing factor to the increasing prevalence of diabetes [73]. Our data suggest that agricultural change and commodification may contribute to this dietary change.

### Food-system differences between oil palm and traditional livelihoods

Agrarian, livelihood, and landscape transitions may alter diets through different pathways at different scales [12,28]. At an individual or household level, diets may be mediated by subsistence food production, income, gender dynamics, and time allocation [15,74], leading to different priorities, preferences, and opportunities for food acquisition. At the food-system level, markets respond to altered supply and demand dynamics, resulting from changes in local production as well as broader social and cultural shifts in food desirability [27,45].

Although we cannot make causal claims, differences in food acquisition patterns between sites allow us to infer differences in food systems. In both sites, markets play a greater role in food acquisition in OP than in TL households, with the most pronounced differences for meat, fish, and vegetables. The types of foods available in market systems mediate the effects of increased reliance on purchased foods. For instance, in WK, a significant proportion of purchased meat was processed, which was not consumed at all in Papua. In both sites, the diversity of purchased meat varieties was relatively low, dominated by chicken and with little bushmeat.

This study also highlights the central role that gifted foods play within local food systems. Food-sharing networks can contribute positively toward food security [75] and are integral components of many traditional food systems [76]. In addition, they increase resilience to climatic and economic shocks [[77], [78], [79], [80]]. In both sites in this study, gifted foods contributed a significant proportion of the meat, fruit, and tubers consumed by children, but in different ways. In Papua, gifts were a significantly greater proportion of these foods in OP than in TL, whereas in WK, the proportion was lower in OP than in TL.

Our data do not identify the original source of gifted foods (i.e., whether agricultural, wild, or purchased). However, it is evident from the types of food that wild and semicultivated varieties comprised a significant component of gifted foods. Such foods are often culturally valued and perceived as healthier and tastier to market alternatives [81,82]. Studies in WK and Papua indicate persistence (and even magnification) of wild food preferences among oil palm workers who engage in wild food acquisition less frequently [45,83]. For some foods, especially sago in Papua, sharing them is integral to cultural identity [83,84]. We hypothesize, therefore, that foods obtained as gifts in the OP site in Papua may originate from kin-relations with more traditional livelihoods. The purpose of these gifts—whether as economic support or due to their lack of availability in local markets—remains unclear.

In contrast to Papua, gifted wild and agricultural foods in WK are common in TL but rare in OP. An associated study in WK posits that oil palm labor is incompatible with producing large surpluses of wild and agricultural foods—and that these foods are those typically gifted in an “informal hyper-local food system of reciprocal gift-giving” [33,45]. Such food-system level differences may be an example of nonlinear system effects of agricultural intensification [27,85]. Although individually, commercialization may improve market access, when widely adopted, it may also alter the composition of market foods available for purchase.

### Dietary transitions and trade-offs

Recent studies of the dietary impacts of agricultural transitions have emphasized the fact that such transitions may be associated with dietary trade-offs [6,18], as well as the need to move beyond simplistic aggregate metrics and explicitly examine both healthy and unhealthy food consumption [49,86]. Our study strongly supports these calls for nuanced approaches, finding clear trade-offs in terms of food groups. To date, most of the literature has used proxies for dietary quality or household food security based on dietary diversity scores. Such metrics are proxies for nutrient adequacy, but do not capture overnutrition [38], changes in UPF consumption, or decreases in fiber consumption. They also fail to capture changes in the quantities of consumption of important food groups. Their use is likely, therefore, to miss important nutritional impacts of dietary changes and trade-offs [49].

Although we cannot make causal inferences from this study, the associations between oil palm engagement and UPF consumption observed here are in line with recent studies, which show associations between increased consumption of UPFs and agricultural commercialization in other contexts [18]. These findings strengthen calls for a greater examination of dietary trade-offs of commercialization, particularly in contexts where nutrition and food-system transitions are occurring and where the burden of diet-related NCDs is increasing.

The results for the differences in diet are consistent with the hypothesis that different land use and livelihood strategies are associated with different dietary patterns. This was true for both the WK and the Papuan sites in our study. We find that several of these differences, particularly in the WK site, are consistent with what has been described as a “dietary transition” [87], in which diets shift from foods that are relatively high in fiber and micronutrients to more highly processed foods, and those that are high in sugar, fat, and salt. Our finding that OP households in WK had higher intakes of dairy and eggs is also characteristic of dietary transition dynamics. However, it is also important to note that not all aspects of the typical dietary transition were observed in our data—for example, we did not find an increased consumption of meat overall, but rather an increased share of meat coming from processed sources.

In WK, the lower consumption of DGLVs among children in OP households, coupled with their higher consumption of processed meats and SSBs, aligns with the broader dietary transition occurring in many parts of Indonesia [49,[88], [89], [90]]. Likewise, the pattern occurring in the Papua site aligns with local dietary transitions reported across Papua and West Papua toward a more “modern Indonesian” diet consisting of fewer tubers (mainly sago) and increased cereals (mainly white rice) and legumes (mainly tofu and tempeh) [73]. Tofu and tempeh are especially characteristic of Javanese diets [49,91], and their spread to other regions of Indonesia has been associated with Javanese transmigration and a homogenization of Indonesian diets toward the dominant Javanese diet [92]. Children in Papua also ate more UPF refined grain products, consistent with dietary transitions observed globally.

Although oil palm appears to be associated with the broader local and regional dietary transitions, the exact nature of the transition is also specific to the local context. For example, although the broader Indonesian nutrition transition is associated with higher fish intake in many places [49,93], we find that oil palm adoption is associated with lower fish consumption in the Papua site, likely because traditional diets are significantly higher in fish than diets in many other regions. Likewise, the nature of staple transitions and their health effects depend greatly on the local context—both in terms of the varieties of rice consumed, and the other risk factors associated with NCDs such as diabetes [[94], [95], [96]].

### Study limitations

This study captures a snapshot of dietary associations within broader, ongoing agrarian and livelihood transitions, centered on the dominant contemporary forms of oil palm engagement in Indonesia’s oil palm frontiers. The associations observed here may not hold as oil palm continues to develop, nor reflect transitions occurring in other oil palm contexts. Likewise, livelihoods in the TL group are dynamic [97] and will continue to adapt to shifts in economic opportunities and changes in landscapes and food systems.

The major limitation of this study is its cross-sectional observational design. Oil palm adoption is not random—either at the community or the household level—and is influenced by a range of socioeconomic, geographic, and political factors, which may also affect diets. We lack historical data on participating households and their previous diets, relying instead on qualitative evidence suggesting that villages and local livelihoods were broadly similar in the past. It is unlikely that endogenous factors are fully accounted for by our control variables. As such, we cannot draw causal inference regarding the association between oil palm adoption and children’s diets, and the TL and OP groups cannot be interpreted as formal counterfactuals. However, by restricting the sample to Indigenous households in communities with historically similar livelihoods before oil palm development in the region, the two groups likely shared similar historical baselines in terms of food systems, food environments, and agrarian practices. Rather than direct comparisons, they provide a structured descriptive comparison, representing plausible sets of local alternative livelihoods.

Our analysis focused on estimating differences in mean food consumption between groups rather than usual intake at the individual level which would require multiple 24-h dietary recalls on nonconsecutive days. Likewise, although 24-h recall multiple-pass dietary recall is well-validated for population-level estimation [63], it may not adequately account for infrequently consumed or seasonal foods. This is particularly a concern in WK, where livelihoods and food systems are centered around swidden cycles. Although in WK, we collected data across two seasons (preharvest and postharvest) to capture some of the seasonal variation, swidden livelihoods are also associated with a range of other seasonal activities, as well as fluctuations in cash flows. In addition, local food systems are responsive to aggregate shifts in food availability and income levels, with markets playing different roles and providing different foods within the food environment throughout the year [45]. OP households in WK also experience fluctuations in food availability and food acquisition behavior as swidden continues to play a (albeit diminished) role in livelihoods and food production, creating periods of intense time scarcity which affect food acquisition behaviors [33]. In addition, diets for OP households are substantially different before and after payday because households experience cyclical (typically biweekly) fluctuations in cash availability and food acquisition behavior––a pattern reflected in cyclical availability of market foods [45].

### Conclusion

This study measured dietary intake among children in Indigenous households in two regions of Indonesia, most closely associated with contemporary oil palm expansion (WK and Papua). In both regions, we compared the diets of children in Indigenous households practicing traditional livelihood strategies with those in Indigenous households engaged in the dominant oil palm system in their respective regions (smallholder “plasma” schemes in WK and plantation labor in Papua).

This study cannot causally attribute these differences between groups to engagement with oil palm alone. Oil palm adoption or nonadoption is not random, and there may be underlying endogeneity that links decisions to engage with oil palm and diets not controlled for in our analysis. Further research is needed using longitudinal study designs to investigate if these changes are attributable to changes in oil palm–based livelihoods. Nevertheless, differences in dietary patterns between children in OP and non-OP households in both regions are characteristic of a dietary transition that trends away from traditional diets toward increased consumption of UPFs. The precise nature of these differences varies by region in ways that reflect ongoing region-specific dietary transitions. Dietary transitions may, depending on context, have positive, negative, or mixed effects on diets. This study found that, in the Papua site, children in OP households consumed smaller quantities of fish and tubers, but larger quantities of cereals, DGLVs, legumes and ultra processed refined grains and bakery products than those in TL households. The associations in WK were more mixed, with children in OP households consuming greater quantities of unhealthy UPFs (processed meat and SSBs), lower quantities of DGLVs, but higher quantities of eggs and dairy—two food groups especially beneficial for the growth and development of young children.

Differences in food sources between the OP and TL groups indicate different food acquisition practices and interactions with local food systems. In both sites, children in OP households consumed a greater proportion of their food from market sources and smaller proportions from agricultural and wild sources. Similar transitions have been observed in other cases of increased market integration [98,99] and may be associated with a broader transition in food systems and food environments [14]. However, it should also be noted that wild sources played an important role in food provision even in market-integrated households in both sites in this study. Access to these foods depends on diverse, multifunctional landscapes and food systems, and may be eroded by increasing homogenization at the landscape and livelihood levels [27].

These findings contribute to a growing body of literature showing that agrarian and livelihood transitions are accompanied by multidimensional changes in diets and food systems. Understanding these transitions requires moving beyond simple assumptions of nutritional improvement or decline and instead examining the dietary trade-offs that accompany changing livelihoods and increasing market integration.

## Consent to participate

Informed consent was obtained from all individual participants included in the study.

## Author contributions

The authors’ responsibilities were as follows – RCP, NNW, EW, ML, MMHU: performed material preparation, data collection, and analysis; AI, DR: wrote the first draft of the manuscript; AI, DR, RCP, BP: contributed to conceptualization; AI: contributed to funding acquisition; AI, RCP, BP, NNW, MMHU: contributed to methodology and methods development; DR: contributed to formal analysis; RCP, NNW, EW, ML: contributed to investigation and project administration; MMHU: contributed to data curation; AI, RCP, NNW, BP: contributed to supervision; and all authors: contributed to the study conception and design, commented on subsequent versions of the manuscript, and read and approved the final manuscript.

## Data availability

Data used in this study are available upon request from CIFOR’s Dataverse Repository https://data.cifor.org/dataverse

## Ethics approval

We obtained permission from Badan Kesatuan Bangsa dan Politik (The National and Political Unity Body) at the provincial and district level, as well as Dinas Kesehatan (the Public Health Office) at district and subdistrict levels to carry out our research. All procedures performed in studies involving human participants were in accordance with the ethical standards of the institutional and national research committees and with the 1964 Helsinki Declaration and its later amendments. The study protocol was approved by the Ethics Committee of the Health Polytechnic Makassar, Indonesia, no. 302/KEPK-PTKMKS/XI/2016 in November 2016 before enumerator recruitment in January 2017. The objectives of the study were explained to all respondents before obtaining their informed written consent. Participants volunteered to join the research study and were informed that their names and other personal information would not be revealed. They were offered a small token gift (a small towel, bag, soap) to thank them for their participation.

## Declaration of Generative AI and AI-assisted technologies in the writing process

No generative AI and AI-assisted technologies were used in preparing the manuscript. During revisions, a general-purpose LLM (Claude Opus 4.6, Anthropic) was used to (i) proofread the manuscript text for grammar and clarity; (ii) check figure labeling/formatting and consistency across the text and figures; (iii) support consistency checks in the reporting of results; and (iv) check formatting of references. After using this tool/service, the author(s) reviewed and edited the content as needed and take(s) full responsibility for the content of the publication.

## Funding

This research was funded by the Drivers of Food Choice (DFC) Competitive Grants Program, which was funded by the UK Government’s Foreign, Commonwealth & Development Office and the Gates Foundation, and managed by the University of South Carolina, Arnold School of Public Health, United States; however, the views expressed do not necessarily reflect the UK Government’s official policies.

## Conflict of interest

The authors report no conflicts of interest.

## Footnotes

1 At the time of the survey, the Papua sites were located in Papua province. However, in 2022, the Indonesian Government changed administrative boundaries and created new provinces in the region. Under today’s administrative map, the provinces are located in Papua and South Papua Provinces.
