# Supplementary material for: Agricultural Commercialization and Dietary Transitions in Traditional Food Systems in Indonesia
Source: Curr Dev Nutr. 2026 Jun 30;10(8):109418. doi: 10.1016/j.cdnut.2026.109418 (PMC13452293; doi:10.1016/j.cdnut.2026.109418)
Supplement: Supplementary file 1 [file mmc1.docx]

# <H1>Appendix A: Construction of Asset Index

<p>Assets used in construction of asset index: boat, car, motorbike, cell phone, television, radio, CD/music player/gas stove, refrigerator/freezer, tablet, and sewing machine.

# <H1>Appendix B: OLS Regressions for Food Group Consumption (Grams)

TABLE B1	Food group consumption (grams/day)—West Kalimantan, OLS (*n* = 963)

|  | Cereals | Dairy | Eggs | Fish | DGLVs | Legumes | Meat | Other veg. | Fruit | Tubers |
| --- | --- | --- | --- | --- | --- | --- | --- | --- | --- | --- |
| Oil palm household | –44.44^1^  [–66.31, –22.56] | 24.56^1^  [10.81, 38.30] | 8.27^2^  [1.31, 15.23] | –3.48  [–11.87, 4.90] | –7.10^1^  [–9.89, –4.31] | 1.56  [–0.42, 3.54] | 0.00  [–4.49, 4.49] | –4.39  [–10.85, 2.06] | 1.04  [–0.42, 2.49] | –1.03  [–4.66, 2.60] |
| Age 2–3 y | –25.75  [–64.70, 13.19] | –2.08  [–23.29, 19.13] | –1.22  [–10.14, 7.69] | 6.48  [–2.46, 15.42] | 1.85  [–0.71, 4.42] | 0.09  [–0.68, 0.85] | 3.25  [–0.72, 7.22] | 0.78  [–6.92, 8.48] | –0.02  [–0.41, 0.36] | –0.58  [–2.73, 1.57] |
| Age 3–4 y | 9.84  [–36.58, 56.27] | –9.17  [–29.80, 11.45] | 0.36  [–7.99, 8.70] | 2.21  [–4.51, 8.93] | 2.89^2^  [0.00, 5.77] | 2.12^2^  [0.36, 3.88] | 3.70^3^  [–0.15, 7.55] | 3.00  [–4.33, 10.34] | 1.10  [–1.04, 3.24] | 0.40  [–2.59, 3.39] |
| Age 4–5 y | 33.38^3^  [–5.85, 72.61] | –10.89  [–31.13, 9.35] | –4.53  [–12.14, 3.07] | 8.11^1^  [2.31, 13.91] | 4.07^2^  [0.84, 7.29] | 2.75^2^  [0.49, 5.01] | 4.61^2^  [0.99, 8.24] | 5.69  [–1.90, 13.27] | 0.26  [–0.52, 1.03] | 3.66  [–1.34, 8.66] |
| Wealth quintile 2 | –21.89  [–51.03, 7.25] | 19.94^2^  [1.26, 38.61] | –1.14  [–7.88, 5.61] | 0.32  [–9.12, 9.75] | 0.22  [–2.93, 3.38] | –1.01  [–5.27, 3.25] | 3.71^2^  [0.30, 7.12] | –1.62  [–9.25, 6.01] | 1.41  [–1.17, 3.98] | 1.79  [–1.54, 5.12] |
| Wealth quintile 3 | –32.71^3^  [–67.54, 2.12] | 10.29  [–2.45, 23.03] | 1.78  [–7.24, 10.81] | –1.47  [–12.58, 9.63] | –0.16  [–4.11, 3.78] | –2.40^3^  [–5.04, 0.24] | 3.34  [–1.69, 8.37] | 9.22  [–3.73, 22.17] | 0.80  [–0.67, 2.28] | –0.07  [–2.21, 2.07] |
| Wealth quintile 4 | –8.13  [–48.76, 32.50] | 22.56^3^  [–0.68, 45.81] | –2.03  [–12.00, 7.94] | –1.04  [–13.04, 10.97] | –1.50  [–5.00, 2.00] | –1.39  [–4.54, 1.75] | 3.42^2^  [0.09, 6.75] | –3.08  [–9.85, 3.69] | 0.11  [–0.21, 0.42] | 4.96  [–2.16, 12.09] |
| Wealth quintile 5 (highest) | –28.78^3^  [–62.93, 5.37] | 28.67^2^  [6.71, 50.63] | 6.13  [–2.85, 15.11] | 3.16  [–9.22, 15.54] | –2.81  [–6.25, 0.63] | –1.74  [–4.55, 1.07] | 8.00^1^  [2.54, 13.47] | 4.21  [–4.75, 13.18] | 0.03  [–0.28, 0.34] | 1.17  [–3.09, 5.42] |
| Sex=female | –20.15^2^  [–36.89, –3.41] | –5.65  [–17.47, 6.17] | –0.48  [–6.58, 5.62] | 1.64  [–4.09, 7.36] | –0.99  [–3.09, 1.11] | 0.15  [–1.93, 2.24] | –1.32  [–4.07, 1.43] | –0.99  [–7.07, 5.09] | 0.85  [–0.41, 2.11] | –1.92  [–4.93, 1.09] |
| Caregiver education: middle | 21.22  [–16.15, 58.59] | 10.71  [–3.86, 25.29] | –0.39  [–7.04, 6.26] | 9.49  [–7.84, 26.82] | –2.97^2^  [–5.44, –0.50] | –0.05  [–1.32, 1.21] | 0.51  [–2.74, 3.77] | 2.98  [–8.68, 14.65] | –0.35  [–0.90, 0.20] | 0.98  [–5.73, 7.69] |
| Caregiver education: secondary | –4.00  [–25.85, 17.86] | –3.09  [–21.29, 15.12] | 9.48^2^  [2.17, 16.79] | 2.88  [–3.69, 9.44] | –1.86  [–4.75, 1.04] | –0.18  [–1.18, 0.81] | –0.69  [–4.88, 3.50] | 2.09  [–6.35, 10.54] | –0.23  [–0.59, 0.13] | –0.13  [–3.75, 3.49] |
| Caregiver from surveyed village | –11.56  [–35.41, 12.30] | –1.35  [–13.58, 10.89] | 0.80  [–4.26, 5.85] | 1.48  [–3.76, 6.72] | –1.16  [–3.68, 1.36] | –0.51  [–2.66, 1.64] | –1.36  [–4.35, 1.63] | 0.15  [–6.71, 7.01] | 0.46  [–0.31, 1.23] | 1.53  [–1.53, 4.60] |
| No. other children in HH | –1.89  [–10.09, 6.30] | –2.92  [–7.42, 1.57] | –0.88  [–4.46, 2.70] | –2.03  [–4.49, 0.42] | 0.09  [–1.07, 1.25] | –0.22  [–0.80, 0.36] | 2.75  [–0.58, 6.09] | –0.01  [–2.98, 2.96] | 0.18  [–0.32, 0.69] | –0.71  [–1.57, 0.15] |
| Postharvest season | –18.04  [–42.03, 5.95] | –18.82^1^  [–32.63, –5.01] | 7.59^1^  [2.56, 12.63] | 8.67^2^  [1.15, 16.20] | –0.88  [–2.82, 1.07] | 2.04^2^  [0.32, 3.76] | 3.34  [–0.81, 7.50] | 11.04^1^  [4.41, 17.67] | 0.75  [–0.43, 1.94] | –5.32^1^  [–8.42, –2.22] |
| Constant | 297.74^1^  [239.80, 355.67] | 22.93^3^  [–1.11, 46.98] | 18.17^1^  [5.54, 30.81] | 13.77^1^  [5.05, 22.49] | 13.82^1^  [9.14, 18.50] | 0.36  [–3.32, 4.04] | –2.02  [–8.73, 4.70] | 5.43  [–1.78, 12.64] | –2.22  [–5.70, 1.25] | 4.37^1^  [1.23, 7.51] |
| Observations | 963 | 963 | 963 | 963 | 963 | 963 | 963 | 963 | 963 | 963 |

OLS coefficients (g/d); 95% confidence intervals in brackets.

Cluster-robust SEs (village level). ^1^*P* < 0.01; ^2^*P* < 0.05; ^3^*P* < 0.10.

Abbreviations: DGLV, dark green leafy vegetable; HH, household; OLS, ordinary least squares.

TABLE B2	Food-group consumption (grams/day)—Papua, OLS (*n* = 345)

|  | Cereals | Dairy | Eggs | Fish | DGLVs | Legumes | Meat | Other veg. | Tubers |
| --- | --- | --- | --- | --- | --- | --- | --- | --- | --- |
| Oil palm household | 54.43^2^  [5.71, 103.16] | 6.13  [–7.30, 19.57] | 2.33  [–2.17, 6.84] | –19.65^1^  [–32.41, –6.90] | 6.76^2^  [1.32, 12.19] | 1.86^2^  [0.13, 3.58] | –2.93  [–8.99, 3.12] | 4.88^3^  [–0.84, 10.60] | –51.29^1^  [–79.21, –23.37] |
| Age 2–3 y | –20.20  [–69.88, 29.47] | –8.92  [–30.02, 12.19] | 2.36^3^  [–0.25, 4.97] | 0.25  [–16.09, 16.58] | –8.36^1^  [–14.24, –2.47] | 0.23  [–0.86, 1.33] | 8.08^2^  [0.98, 15.18] | 1.27  [–3.52, 6.07] | 9.46  [–12.67, 31.58] |
| Age 3–4 y | 22.68  [–31.50, 76.87] | –7.15  [–22.21, 7.92] | 0.64  [–2.90, 4.18] | 7.30  [–14.96, 29.57] | –6.73^2^  [–12.24, –1.22] | 0.20  [–0.74, 1.15] | 2.62  [–2.35, 7.59] | 0.12  [–4.22, 4.46] | 3.36  [–14.62, 21.34] |
| Age 4–5 y | 40.52  [–16.45, 97.49] | –9.39  [–29.10, 10.32] | 3.45  [–2.29, 9.19] | 4.05  [–13.81, 21.92] | –4.79  [–12.60, 3.02] | 1.51  [–0.41, 3.43] | 11.31^2^  [1.31, 21.31] | 3.07  [–2.36, 8.50] | 20.13^3^  [–3.88, 44.15] |
| Wealth quintile 2 | –15.28  [–51.86, 21.31] | 10.27  [–12.47, 33.02] | 3.11  [–2.98, 9.20] | 18.15^3^  [–1.50, 37.81] | 2.11  [–2.62, 6.84] | 0.11  [–1.26, 1.49] | 1.31  [–7.63, 10.25] | –0.47  [–4.61, 3.67] | 16.42^1^  [4.43, 28.41] |
| Wealth quintile 3 | 46.56  [–28.69, 121.82] | –1.35  [–5.37, 2.68] | –0.85  [–5.98, 4.29] | 15.33^2^  [1.78, 28.88] | –1.89  [–10.55, 6.77] | –0.19  [–2.65, 2.26] | –0.06  [–9.76, 9.64] | –2.42  [–6.64, 1.81] | 23.57  [–12.48, 59.62] |
| Wealth quintile 4 | –22.86  [–99.87, 54.14] | 6.18  [–10.06, 22.42] | –1.21  [–4.70, 2.27] | 48.48^2^  [8.61, 88.34] | –0.74  [–10.81, 9.32] | 0.10  [–1.03, 1.23] | –8.28  [–20.67, 4.11] | –0.57  [–5.70, 4.55] | 48.95  [–11.92, 109.82] |
| Wealth quintile 5 (highest) | –2.31  [–79.61, 74.99] | 6.60  [–9.56, 22.76] | 0.80  [–3.40, 5.00] | 28.72^1^  [10.47, 46.98] | –3.48  [–8.32, 1.35] | 1.75  [–1.17, 4.67] | –3.68  [–14.80, 7.43] | 0.30  [–6.19, 6.79] | 8.23  [–14.08, 30.55] |
| Sex=female | –12.48  [–52.73, 27.76] | –3.73  [–13.66, 6.20] | –1.25  [–4.32, 1.82] | 8.41^3^  [–1.15, 17.96] | –4.01  [–9.19, 1.17] | –0.89  [–2.66, 0.88] | 3.65  [–3.84, 11.15] | 3.90^3^  [–0.30, 8.10] | –10.26  [–35.61, 15.08] |
| Caregiver education: middle | 64.49^2^  [5.06, 123.92] | –3.07  [–10.08, 3.94] | –0.55  [–4.11, 3.01] | –3.34  [–20.11, 13.43] | 1.82  [–4.14, 7.79] | 0.18  [–1.54, 1.90] | 1.71  [–6.17, 9.59] | 0.69  [–4.95, 6.34] | –22.14  [–57.49, 13.21] |
| Caregiver education: secondary | 9.13  [–35.46, 53.73] | 4.21  [–6.65, 15.08] | 0.18  [–3.60, 3.97] | 8.49  [–6.16, 23.13] | –0.60  [–6.68, 5.47] | –0.26  [–1.83, 1.31] | –0.19  [–7.12, 6.73] | 0.45  [–5.86, 6.77] | –19.40^3^  [–40.87, 2.07] |
| Caregiver from surveyed village | 17.87  [–21.06, 56.80] | –6.73  [–21.90, 8.43] | –3.61  [–9.39, 2.18] | –0.34  [–12.17, 11.49] | 2.38  [–2.59, 7.35] | –0.27  [–1.84, 1.30] | –2.37  [–8.73, 4.00] | –2.67  [–10.57, 5.22] | 4.85  [–17.56, 27.26] |
| No. other children in HH | –7.07  [–19.54, 5.40] | –1.64  [–4.58, 1.31] | –1.05^2^  [–1.90, –0.20] | 0.69  [–2.72, 4.09] | –1.25^1^  [–2.02, –0.47] | –0.22  [–0.75, 0.32] | –0.46  [–3.51, 2.60] | 0.87  [–0.40, 2.14] | 4.09  [–1.67, 9.86] |
| Ethnicity: Yei | 34.01  [–23.36, 91.38] | 0.99  [–1.82, 3.80] | 2.60^3^  [–0.29, 5.49] | 2.54  [–15.29, 20.36] | 8.11^1^  [2.30, 13.93] | 0.06  [–1.32, 1.44] | –4.62^3^  [–9.41, 0.16] | 5.12  [–6.59, 16.83] | –41.01^2^  [–78.05, –3.96] |
| Ethnicity: other | 43.97^3^  [–4.69, 92.63] | 8.98  [–6.29, 24.26] | 3.46  [–4.15, 11.08] | –10.22^3^  [–21.44, 1.00] | 6.94^3^  [–1.15, 15.04] | 0.53  [–1.38, 2.44] | –1.77  [–7.51, 3.96] | 5.64^2^  [1.43, 9.85] | –32.08^1^  [–53.69, –10.47] |
| Constant | 121.18^2^  [31.69, 210.66] | 9.34  [–9.46, 28.15] | 3.67  [–4.93, 12.26] | 22.01  [–8.88, 52.90] | 15.51^1^  [6.74, 24.29] | 0.11  [–2.52, 2.75] | 5.58  [–4.92, 16.08] | –3.20  [–9.05, 2.65] | 65.20^2^  [4.95, 125.44] |
| Observations | 345 | 345 | 345 | 345 | 345 | 345 | 345 | 345 | 345 |

OLS coefficients (g/d); 95% confidence intervals in brackets.

Cluster-robust SEs (village level). ^1^*P* < 0.01; ^2^*P* < 0.05; ^3^*P* < 0.10.

Abbreviations: DGLV, dark green leafy vegetable; HH, household; OLS, ordinary least squares.

# <H1>Appendix C: Logit Regressions for Processed Food Consumption

TABLE C1	Processed-food consumption—West Kalimantan, logit odds ratios (*n* = 963)

|  | All UPFs | SSBs | Sweets | Refined grains | Processed meat |
| --- | --- | --- | --- | --- | --- |
| Oil palm household | 1.130  [0.833, 1.533] | 1.742^1^  [1.182, 2.569] | 1.064  [0.811, 1.398] | 0.965  [0.733, 1.270] | 4.021^1^  [1.612, 10.028] |
| Age 2–3 y | 1.420  [0.880, 2.291] | 1.064  [0.600, 1.886] | 1.400  [0.885, 2.216] | 1.581^2^  [1.026, 2.436] | 1.849  [0.673, 5.081] |
| Age 3–4 y | 1.765^2^  [1.138, 2.737] | 1.238  [0.746, 2.056] | 1.283  [0.784, 2.099] | 1.932^1^  [1.351, 2.762] | 2.064^3^  [0.906, 4.701] |
| Age 4–5 y | 2.166^1^  [1.394, 3.364] | 1.457  [0.835, 2.541] | 1.294  [0.800, 2.092] | 2.259^1^  [1.542, 3.310] | 2.796^2^  [1.228, 6.367] |
| Wealth quintile 2 | 1.534^3^  [0.937, 2.514] | 1.957^1^  [1.292, 2.963] | 1.645^2^  [1.078, 2.508] | 1.038  [0.740, 1.458] | 1.014  [0.493, 2.084] |
| Wealth quintile 3 | 1.148  [0.745, 1.770] | 1.422  [0.880, 2.297] | 1.234  [0.839, 1.814] | 0.919  [0.543, 1.554] | 0.944  [0.334, 2.665] |
| Wealth quintile 4 | 1.707^2^  [1.010, 2.883] | 1.472  [0.787, 2.756] | 1.170  [0.758, 1.808] | 2.122^1^  [1.389, 3.241] | 1.442  [0.604, 3.441] |
| Wealth quintile 5 (highest) | 1.447  [0.903, 2.319] | 1.406  [0.833, 2.373] | 1.664^2^  [1.098, 2.522] | 1.116  [0.726, 1.717] | 1.397  [0.658, 2.968] |
| Sex=female | 0.990  [0.749, 1.310] | 1.117  [0.781, 1.597] | 1.124  [0.878, 1.439] | 0.973  [0.772, 1.226] | 1.250  [0.732, 2.136] |
| Caregiver education II | 1.105  [0.748, 1.633] | 0.831  [0.499, 1.384] | 1.177  [0.864, 1.604] | 0.958  [0.616, 1.489] | 1.860^3^  [0.993, 3.485] |
| Caregiver education III | 1.195  [0.898, 1.589] | 0.819  [0.469, 1.431] | 1.089  [0.817, 1.451] | 0.933  [0.684, 1.272] | 0.970  [0.456, 2.061] |
| Caregiver from surveyed village | 1.146  [0.838, 1.566] | 1.218  [0.843, 1.759] | 1.130  [0.847, 1.509] | 1.072  [0.814, 1.411] | 1.264  [0.563, 2.835] |
| No. other children in HH | 0.916  [0.790, 1.061] | 0.902  [0.711, 1.145] | 0.916  [0.816, 1.028] | 0.970  [0.828, 1.135] | 1.171  [0.856, 1.600] |
| Postharvest season | 1.189  [0.824, 1.716] | 1.411^3^  [0.947, 2.101] | 0.924  [0.699, 1.223] | 0.936  [0.704, 1.244] | 0.808  [0.457, 1.429] |
| Observations | 963 | 963 | 963 | 963 | 963 |

Odds ratios from logistic regression; 95% confidence intervals in brackets.

Cluster-robust SEs (village level). ^1^*P* < 0.01; ^2^*P* < 0.05; ^3^*P* < 0.10.

Abbreviations: HH, household; SSB, sugar-sweetened beverage; UPF, ultraprocessed food.

TABLE C2	Processed-food consumption—Papua, logit odds ratios (*n* = 345)

|  | All UPFs | SSBs | Sweets | Refined grains |
| --- | --- | --- | --- | --- |
| Oil palm household | 1.823  [0.868, 3.828] | 1.794  [0.519, 6.203] | 1.614  [0.780, 3.338] | 2.660^2^  [1.234, 5.731] |
| Age 2–3 y | 3.006^1^  [1.615, 5.595] | 0.907  [0.259, 3.183] | 3.048^1^  [1.704, 5.450] | 1.742  [0.520, 5.835] |
| Age 3–4 y | 2.576^1^  [1.534, 4.325] | 2.281  [0.520, 10.011] | 2.474^1^  [1.397, 4.384] | 3.536^2^  [1.213, 10.308] |
| Age 4–5 y | 1.936^2^  [1.022, 3.668] | 0.822  [0.245, 2.753] | 1.641  [0.764, 3.525] | 2.711^1^  [1.296, 5.674] |
| Wealth quintile 2 | 1.290  [0.546, 3.050] | 1.733  [0.574, 5.239] | 2.564^2^  [1.014, 6.482] | 0.795  [0.346, 1.825] |
| Wealth quintile 3 | 1.158  [0.464, 2.890] | 1.671  [0.468, 5.969] | 1.222  [0.527, 2.834] | 0.749  [0.262, 2.144] |
| Wealth quintile 4 | 0.961  [0.342, 2.697] | 1.000  [1.000, 1.000] | 2.136  [0.472, 9.655] | 0.724  [0.212, 2.478] |
| Wealth quintile 5 (highest) | 2.052  [0.765, 5.506] | 1.247  [0.276, 5.644] | 4.228^1^  [1.414, 12.644] | 0.767  [0.336, 1.751] |
| Sex=Female | 0.952  [0.594, 1.527] | 1.524  [0.476, 4.878] | 1.224  [0.780, 1.918] | 0.585  [0.257, 1.329] |
| Caregiver education: middle | 1.857^2^  [1.031, 3.346] | 1.156  [0.315, 4.237] | 1.879^3^  [0.949, 3.721] | 1.684^3^  [0.952, 2.979] |
| Caregiver education: secondary | 1.129  [0.569, 2.240] | 0.411^1^  [0.214, 0.790] | 1.093  [0.631, 1.896] | 1.346  [0.675, 2.683] |
| Caregiver from surveyed village | 0.479^1^  [0.287, 0.798] | 0.321^1^  [0.144, 0.715] | 0.687  [0.416, 1.135] | 0.695  [0.335, 1.440] |
| No. other children in HH | 0.880^2^  [0.774, 1.000] | 0.880  [0.714, 1.084] | 0.917  [0.803, 1.047] | 0.729^1^  [0.594, 0.894] |
| Ethnicity: Yei | 0.915  [0.389, 2.155] | 1.287  [0.349, 4.740] | 1.007  [0.496, 2.042] | 1.297  [0.472, 3.566] |
| Ethnicity: other | 0.860  [0.530, 1.393] | 1.373  [0.446, 4.225] | 1.100  [0.634, 1.908] | 0.715  [0.332, 1.540] |
| Observations | 345 | 329 | 345 | 345 |

Odds ratios from logistic regression; 95% confidence intervals in brackets.

Cluster-robust SEs (village level). ^1^*P* < 0.01; ^2^*P* < 0.05; ^3^*P* < 0.10.

Abbreviations: HH, household; SSB, sugar-sweetened beverage; UPF, ultraprocessed food.

**<H1>Appendix D: Sensitivity Analysis**

TABLE D1	Log-transformed outcomes ln(x+1)—West Kalimantan, OLS (*n* = 963)

|  | Cereals | Dairy | Eggs | Fish | DGLVs | Legumes | Meat | Other veg. | Fruit | Tubers |
| --- | --- | --- | --- | --- | --- | --- | --- | --- | --- | --- |
| Oil palm household | –0.290^1^  (0.050) | 0.577^1^  (0.134) | 0.464^2^  (0.182) | –0.349^2^  (0.176) | –0.697^1^  (0.131) | 0.088  (0.054) | 0.219  (0.182) | –0.137  (0.088) | 0.027  (0.018) | –0.117^2^  (0.050) |
| Age 2–3 y | –0.033  (0.126) | –0.363^3^  (0.212) | –0.013  (0.204) | 0.004  (0.181) | 0.112  (0.099) | 0.072^3^  (0.041) | 0.249^3^  (0.142) | –0.034  (0.185) | 0.001  (0.005) | 0.001  (0.050) |
| Age 3–4 y | 0.167  (0.115) | –0.499^2^  (0.228) | 0.037  (0.208) | –0.120  (0.171) | 0.210  (0.133) | 0.192^1^  (0.055) | 0.227^2^  (0.106) | 0.177  (0.142) | 0.021  (0.02) | 0.014  (0.057) |
| Age 4–5 y | 0.188^3^  (0.100) | –0.583^1^  (0.205) | –0.213  (0.190) | 0.255^3^  (0.147) | 0.231^3^  (0.135) | 0.184^1^  (0.056) | 0.292^2^  (0.118) | 0.288^2^  (0.145) | 0.014  (0.016) | 0.067  (0.065) |
| Wealth quintile 2 | –0.024  (0.056) | 0.506^1^  (0.168) | 0.056  (0.170) | 0.198  (0.146) | 0.024  (0.116) | –0.081  (0.081) | 0.307^2^  (0.126) | –0.139  (0.125) | 0.027  (0.024) | 0.046  (0.058) |
| Wealth quintile 3 | –0.073  (0.074) | 0.407^2^  (0.172) | 0.140  (0.214) | 0.016  (0.226) | 0.002  (0.155) | –0.117^3^  (0.066) | 0.259  (0.187) | 0.150  (0.148) | 0.034  (0.033) | –.001  (0.061) |
| Wealth quintile 4 | –0.105  (0.110) | 0.494^2^  (0.223) | 0.012  (0.263) | 0.240  (0.204) | –0.135  (0.130) | –0.030  (0.080) | 0.290^3^  (0.157) | –0.025  (0.165) | 0.003  (0.004) | 0.019  (0.077) |
| Wealth quintile 5 (highest) | –0.144^3^  (0.078) | 0.720^1^  (0.202) | 0.304  (0.204) | 0.209  (0.210) | –0.173  (0.154) | –0.034  (0.082) | 0.569^1^  (0.194) | 0.130  (0.146) | 0.002  (0.004) | 0.023  (0.084) |
| Sex=female | –0.111^2^  (0.048) | –.031  (0.118) | –0.032  (0.144) | 0.124  (0.121) | –0.100  (0.106) | 0.021  (0.049) | –.109  (0.078) | –.005  (0.088) | 0.023  (0.016) | –0.082^3^  (0.048) |
| Caregiver education II | 0.007  (0.079) | 0.406^1^  (0.156) | 0.283  (0.201) | 0.303^2^  (0.143) | –0.168^3^  (0.096) | 0.064  (0.074) | 0.041  (0.091) | 0.117  (0.120) | –0.009  (0.007) | –0.097  (0.073) |
| Caregiver education III | –0.017  (0.060) | –0.011  (0.175) | 0.465^1^  (0.178) | 0.187  (0.180) | –0.072  (0.149) | –.003  (0.047) | –0.100  (0.152) | –0.079  (0.148) | –.006  (0.004) | –0.076  (0.066) |
| Caregiver from surveyed village | –0.092  (0.066) | –0.052  (0.121) | 0.158  (0.137) | 0.074  (0.100) | –0.155^3^  (0.088) | –0.049  (0.055) | –0.105  (0.116) | 0.074  (0.102) | 0.012  (0.009) | 0.046  (0.046) |
| No. other children in HH | –.006  (0.023) | 0.003  (0.052) | –0.050  (0.095) | –0.107^3^  (0.062) | –.014  (0.054) | –0.017  (0.014) | 0.181^3^  (0.096) | –0.034  (0.045) | 0.002  (0.005) | –0.024  (0.018) |
| Post–harvest season | –0.133^2^  (0.052) | –0.199^3^  (0.106) | 0.154  (0.132) | 0.115  (0.138) | –0.131  (0.103) | 0.105^2^  (0.043) | 0.004  (0.134) | 0.305^1^  (0.114) | 0.019  (0.014) | –0.214^1^  (0.044) |
| Constant | 5.508^1^  (0.138) | 0.552^2^  (0.247) | 1.257^1^  (0.330) | 1.565^1^  (0.258) | 1.892^1^  (0.216) | 0.009  (0.088) | 0.065  (0.195) | 0.771^1^  (0.202) | –.055  (0.039) | 0.344^1^  (0.089) |
| Observations | 963 | 963 | 963 | 963 | 963 | 963 | 963 | 963 | 963 | 963 |
| *R²* | 0.055 | 0.068 | 0.027 | 0.031 | 0.069 | 0.028 | 0.040 | 0.029 | 0.013 | 0.032 |

OLS coefficients on log-transformed outcomes, ln(x+1); robust SEs in parentheses. ^1^*P* < 0.01; ^2^*P* < 0.05; ^3^*P* < 0.10.

Abbreviations: DGLV, dark green leafy vegetable; HH, household; OLS, ordinary least squares.

TABLE D2	Log-transformed outcomes ln(*x* + 1)—Papua, OLS (*n* = 345)

|  | Cereals | Dairy | Eggs | Fish | DGLVs | Legumes | Meat | Other veg. | Tubers |
| --- | --- | --- | --- | --- | --- | --- | --- | --- | --- |
| Oil palm household | 1.322^1^  (0.334) | 0.046  (0.070) | 0.310^3^  (0.174) | –0.644^1^  (0.248) | 0.260  (0.239) | 0.198^1^  (0.074) | 0.033  (0.201) | 0.268  (0.188) | –1.526^1^  *(0.361)* |
| Age 2–3 y | –0.418  (0.317) | –0.088  (0.142) | 0.157  (0.142) | 0.216  (0.230) | –0.540^2^  (0.231) | 0.034  (0.063) | 0.513^2^  (0.214) | 0.111  (0.203) | 0.397^2^  *(0.199)* |
| Age 3–4 y | –0.048  (0.369) | –0.070  (0.079) | 0.026  (0.179) | 0.147  (0.292) | –0.450^2^  (0.209) | 0.019  (0.060) | 0.394^3^  (0.210) | –0.021  (0.130) | 0.106  *(0.191)* |
| Age 4–5 y | 0.045  (0.291) | –0.134  (0.120) | 0.213  (0.174) | 0.289  (0.326) | –0.496^2^  (0.248) | 0.120  (0.075) | 0.523^2^  (0.202) | 0.153  (0.120) | 0.338  *(0.213)* |
| Wealth quintile 2 | –0.453^2^  (0.184) | 0.117  (0.143) | 0.063  (0.172) | 0.747^2^  (0.324) | 0.039  (0.191) | –.007  (0.073) | 0.107  (0.218) | –0.076  (0.170) | 0.701^1^  *(0.210)* |
| Wealth quintile 3 | –0.087  (0.274) | –0.028  (0.049) | –0.149  (0.251) | 0.787^3^  (0.402) | 0.01  (0.358) | –0.035  (0.108) | 0.091  (0.25) | –0.013  (0.201) | 0.609^2^  *(0.297)* |
| Wealth quintile 4 | –0.120  (0.499) | 0.036  (0.106) | –0.216  (0.138) | 1.950^1^  (0.629) | –0.279  (0.463) | 0.001  (0.054) | –0.278  (0.332) | –0.244  (0.193) | 1.456^1^  *(0.427)* |
| Wealth quintile 5 (highest) | –0.230  (0.357) | 0.125  (0.172) | –0.181  (0.122) | 1.417^1^  (0.350) | –0.107  (0.272) | 0.140  (0.120) | 0.119  (0.340) | 0.018  (0.182) | 0.569^2^  *(0.287)* |
| Sex=Female | –0.107  (0.224) | –.016  (0.077) | –0.151  (0.114) | 0.189  (0.195) | –0.278^2^  (0.135) | –0.088  (0.071) | –0.029  (0.145) | 0.186  (0.136) | –0.051  *(0.221)* |
| Caregiver education: middle | 1.017^1^  (0.264) | –0.063  (0.095) | 0.026  (0.151) | –0.223  (0.300) | 0.107  (0.219) | 0.031  (0.087) | 0.288  (0.226) | 0.255  (0.201) | –0.575^2^  *(0.279)* |
| Caregiver education: secondary | 0.543^3^  (0.322) | –.019  (0.049) | –0.065  (0.123) | 0.389  (0.256) | 0.059  (0.219) | –0.034  (0.060) | 0.261^3^  (0.137) | 0.024  (0.187) | –0.171  *(0.275)* |
| Caregiver from surveyed village | 0.227  (0.279) | –0.022  (0.080) | –0.237  (0.170) | 0.121  (0.265) | 0.104  (0.193) | –0.042  (0.069) | –0.244^3^  (0.146) | –0.043  (0.193) | 0.263  *(0.237)* |
| No. other children in HH | –0.184^2^  (0.083) | –0.040  (0.029) | –0.083^1^  (0.031) | 0.042  (0.066) | –0.110^1^  (0.032) | –0.013  (0.023) | 0.016  (0.058) | 0.052  (0.053) | 0.147^1^  *(0.054)* |
| Ethnicity: Yei | 0.900^2^  (0.448) | 0.011  (0.023) | 0.232^3^  (0.120) | 0.309  (0.327) | 0.845^1^  (0.174) | 0.051  (0.087) | –0.182  (0.202) | 0.132  (0.205) | –1.180^2^  *(0.560)* |
| Ethnicity: other | 0.981^1^  (0.318) | 0.219  (0.136) | 0.110  (0.219) | –0.482^3^  (0.247) | 0.471^2^  (0.238) | 0.034  (0.096) | 0.007  (0.212) | 0.487^1^  (0.154) | –1.069^1^  *(0.289)* |
| Constant | 3.366^1^  (0.584) | 0.125  (0.125) | 0.450^3^  (0.257) | 1.219^1^  (0.400) | 1.965^1^  (0.385) | 0.028  (0.117) | 0.160  (0.344) | –.109  (0.252) | 1.736^1^  *(0.650)* |
| Observations | 345 | 345 | 345 | 345 | 345 | 345 | 345 | 345 | 345 |

OLS coefficients on log-transformed outcomes, ln(*x*+1); robust SEs in parentheses. ^1^*P* < 0.01; ^2^*P* < 0.05; ^3^*P* < 0.10.

Abbreviations: DGLV, dark green leafy vegetable; HH, household; OLS, ordinary least squares.

**<H1>Appendix E: Supplementary Methods**

<H2>Enumerator recruitment, training, and field testing

<p>The study protocol was approved on 3 November, 2016, by the Ethics Committee of the Health Polytechnic Makassar, Indonesia, no. 302/KEPK-PTKMKS/XI/2016. Enumerator recruitment began on 31 January, 2017.

<p>We partnered with the provincial branches of the polytechnic schools of nutrition in the 2 provinces where the study was conducted—Poltekkes Pontianak and Poltekkes Jayapura. The head of the nutrition department at each institution recruited 10 recent graduates to serve as enumerators for the project. Enumerators received training from the project team on 24-h dietary recall data collection methods, as well as the administration of the questionnaire’s socioeconomic and agricultural modules. The training took place over 4 d in each site and was followed by 2 d of pretesting the instruments and of observing the enumerators by 2 supervisors. After the pretest, the questionnaire underwent minor revisions to better reflect the local context for each site. During training for dietary intake, the topics covered included how to develop rapport with respondents, multiple-pass methods, how to probe without leading the respondent, and how to estimate portion sizes using playdough.

<H2>Data collection and supervision

<p>The data collection team consisted of 2 supervisors and 10 enumerators. In each study area (subdistrict) in West Kalimantan and Papua, 1 supervisor was assigned to oversee 5 enumerators throughout the data collection period. Enumerators conducted interviews with female caregivers either in their homes or at designated community health posts. Enumerators visited households in pairs, with 1 enumerator tasked with leading the interview and the other with additional probing and overseeing data completeness. At the end of each day, supervisors were responsible for ensuring the completeness and accuracy of the 24-h dietary recall questionnaires. Their tasks included verifying the chronologic sequence of food consumption within the recall period, checking the consistency and continuity of linked questions (e.g., from mixed dishes and recipes to individual ingredients, quantities, and units), and identifying any missing food intake information. Errors, inconsistencies, or missing information were identified during the supervisory review and, where necessary, enumerators returned to respondents to verify and collect the missing information, ensuring the quality and completeness of the dataset.

<p>Supervisors also reviewed the entirety of the completed questionnaires to ensure their completeness and accuracy. Particular attention was given to variables involving linked questions, as well as data required for the assessment of nutritional status, including the child’s sex, date of birth, date of survey, body weight, and height/length. Any inconsistencies, missing values, or recording errors were corrected before the questionnaires were submitted for data entry and analysis.

<p>In addition, 1 supervisor was assigned to manage the updating of the food conversion database by collecting samples of newly reported foods, weighing them, recording the measurements, and calculating the corresponding conversion factors. All foods reported in the 24-h dietary recall were converted into grams before data entry.

<H2>Food weighing and conversions

<p>Dietary recall data were processed in Stata 17 to estimate the actual amount of each food ingredient consumed by respondents. For nonrecipe foods, the reported edible portion weight was directly used as the final consumption weight. For recipe-based foods, ingredient weights were adjusted using yield factors to account for changes in food weight during preparation and cooking. For boiled foods, an additional water transformation factor was applied to account for weight changes resulting from water absorption during the boiling process. The resulting values represented the estimated weight of each food ingredient consumed by the respondent.

<H3>***Local household unit and food conversion study methodology.***

<p>Before the dietary survey, a study of local household units and food conversion factors was conducted at both study sites (WK and Papua) with the following 2 objectives: *1*) to convert local household food measures into standard international units (grams and liters) and *2*) to develop a food conversion database that could be used by enumerators to estimate food consumption during data collection.

<p>The food conversion study was conducted by 3 professional nutritionists in WK and Papua before the main survey. We initially performed key informant interviews with 7 respondents in WK and 3 in Papua. During data collection, the database was continuously expanded and updated with support from the 10 enumerators.

<p>The food conversion database included the following components:

<p>1. Household utensil conversions

Commonly used local household utensils in WK and Papua were collected, including different types, sizes, and materials of plates, bowls, glasses, pans, soup spoons, and other serving utensils. Each utensil was filled with water and weighed 5 times. The average weight or volume was recorded and used as the conversion factor for that utensil.

<p>2. Spoon conversions

Different types, sizes, and materials of spoons were filled with sugar and weighed 5 times. The average weight was recorded to establish standard conversion factors for spoon measures.

<p>3. Carbohydrate sources

Various cereal-based foods reported by respondents were collected in both raw and cooked forms. Different portion sizes were weighed 5 times, and the average weight for each portion size was recorded. Different sizes and portions of tubers (e.g., potatoes and sweet potatoes) were collected and weighed. For each size category, corresponding playdough replicas were created. Conversion factors were calculated by dividing the weight of the actual tuber by the weight of the playdough replica.

<p>4. Meat and fish

Various cooked meat portions and fish fillets were collected. Each portion size was weighed 5 times, and corresponding playdough replicas were produced. Conversion factors were calculated by dividing the weight of the actual food by the weight of the playdough replica.

<p>5. Eggs

Different types and sizes of eggs (e.g., chicken eggs and quail eggs) were collected. Raw and cooked eggs were weighed 5 times, and the average weight was recorded as the standard household unit for each egg type and size.

<p>6. Plant-based protein sources

Various portion sizes of tofu and tempeh were collected and weighed 5 times. Corresponding playdough replicas were created, and conversion factors were calculated by dividing the weight of the actual food by the weight of the playdough replica.

<p>7. Vegetables

Commonly consumed vegetables in the study areas, including leafy greens, wild vegetables, and mushrooms, were collected. Vegetables were placed into different household containers or measured as local units (e.g., one bundle/ikat). Each measurement was weighed 3 times, and the average weight was recorded.

<p>8. Fruits

Various fruit types and sizes were collected whenever possible. Conversion factors were established using one of the following methods: weighing each size of fruit 3 times and recording the average weight; direct weighing of individual fruits; or creating playdough replicas and calculating conversion factors based on the ratio of actual fruit weight to replica weight.

<p>9. Beverages, milk, dairy products, and oils

For homemade beverages, milk and dairy products, tea, coffee, and cooking oil, water-based volume conversion methods were used to estimate standard household measures.

<p>10. Natural spices and condiments

Commonly consumed spices and condiments (e.g., tamarind, bilimbi, pepper, and chili) were collected in raw form and weighed 3 times. The average weight was recorded as the standard household unit.

<p>11. Homemade snacks

Various homemade snacks (e.g., fried tofu and fried tempeh) were collected in different portion sizes. Each portion was weighed 3 times and compared with corresponding playdough replicas to establish conversion factors.

<p>12. Commercially produced foods

Commonly consumed packaged foods (e.g., instant noodles, chips, biscuits, and confectionery products) were collected from respondents or local kiosks. Product names, food categories, package sizes, and weights were recorded directly from packaging information.

<H2>Validation of 24-h recall

<p>To ensure that our 24-h recall methods were accurately capturing consumption patterns, we performed a small validation study in the Kapuas Hulu site with 14 adult female respondents. Enumerators observed their food consumption and weighed their foods over a 1-d period, with different enumerators conducting a 24-h recall with the same respondents on the following day. The results showed no statistically significant differences in quantities between the 2 methods (Table E1).

TABLE E1	Comparative analysis of intakes from 24-h recall and direct food weighing (*n* = 14, Kapuas Hulu, West Kalimantan)

| Variables | Indonesia (*n* = 14) | | | | | |
| --- | --- | --- | --- | --- | --- | --- |
|  | 24-h recall | | Food weighing | | *P* value | |
|  | Mean ± SD | Median (Q1, Q3) | Mean ± SD | Median (Q1, Q3) | t-test | Wilcoxon rank |
| Total intake | 674.3 ± 238.6 | 640.3 (542.5, 749.1) | 655.7 ± 223.8 | 638.8 (490.0, 837.0) | NS | NS |
| Staple | 449.4 ± 166.9 | 408.0 (337.0, 431.0) | 422.0 ± 146.1 | 414.0 (311.0, 448.0) | NS | NS/< |
| Nonstaple | 224.8 ± 141.2 | 231.3 (135.5, 314.0) | 233.7 ± 151.3 | 243.0 (124.0, 366.0) | NS/< | NS/< |
| Vegetable | 21.1 ± 34.9 | 0.0 (0.0, 69.0) | 20.4 ± 34.1 | 0.0 (0.0, 55.0) | NS | NS |
| Fruit | 5.7 ± 21.4 | 0.0 (0.0, 0.0) | 5.7 ± 21.4 | 0.0 (0.0, 0.0) | NS/= | NS/= |
| ASF | 68.9 ± 66.6 | 56.5 (0.0, 110.0) | 74.8 ± 77.7 | 54.5 (128.0) | NS/< | NS |
| Oil | 4.4 ± 3.0 | 4.0 (2.5, 6.6) | 3.0 ± 3.0 | 2.5 (0.0, 5.0) | NS | NS |

Abbreviation: NS, not significant.

<H2>Focus group discussions

<p>Twenty focus group discussions (FGDs) were conducted in WK, and 10 FGDs were conducted in Papua. We also conducted in-depth interviews to improve data reliability through triangulation. During the FGDs, we conducted mapping exercises, collected seasonal calendars, and collected lists of wild foods in each village. FGDs were carried out separately with males and females. Using an open-ended interview protocol to guide discussion, facilitators with good command of local languages were supervised by a qualitative researcher. Interview questions were developed to gather information related to dietary habits, including frequently eaten and wild foods, access to and sources of food, and perceptions of dietary quality. Data were analyzed using descriptive thematic analysis. Interviews were audio recorded and transcribed. Transcripts were sorted and coded to determine themes relating to the research questions.
